# Similarities and differences between HIV and SARS-CoV-2

**DOI:** 10.7150/ijms.50133

**Published:** 2021-01-01

**Authors:** Francisco Illanes-Álvarez, Denisse Márquez-Ruiz, Mercedes Márquez-Coello, Sara Cuesta-Sancho, José Antonio Girón-González

**Affiliations:** Unidad de Enfermedades Infecciosas, Servicio de Medicina Interna, Hospital Universitario Puerta del Mar, Facultad de Medicina, Universidad de Cádiz, Cádiz, Spain. Instituto de Investigación e Innovación en Ciencias Biomédicas de la Provincia de Cádiz (INiBICA).

**Keywords:** COVID-19, AIDS, pandemics, HIV, SARS-CoV-2

## Abstract

In the last 50 years we have experienced two big pandemics, the HIV pandemic and the pandemic caused by SARS-CoV-2.

Both pandemics are caused by RNA viruses and have reached us from animals. These two viruses are different in the transmission mode and in the symptoms they generate. However, they have important similarities: the fear in the population, increase in proinflammatory cytokines that generate intestinal microbiota modifications or NETosis production by polymorphonuclear neutrophils, among others. They have been implicated in the clinical, prognostic and therapeutic attitudes.

## Introduction

In the last 50 years, we have experienced two pandemics, the human immunodeficiency virus (HIV) and the severe acute respiratory syndrome coronavirus 2 (SARS-CoV-2) infections.

Both are caused by natural viruses that have reached us from animals 1-3. Although these two viruses are different in the transmission mode and in the symptoms they generate, they have important similarities between them.

HIV infection was discovered in the 80s (1981), when the first cases were known in young adults in California. It is believed that HIV spread to humans through direct contact with the infected blood of chimpanzees by hunting them for their meat. They were affected by severe pneumonia caused by *Pneumocystis jiroveci* or with Kaposi's sarcoma. The transmission mechanism was sexual contact, drugs injection, blood transfusions and mother-to-child. A social stigma was generated. HIV spread rapidly throughout the world and the death rate was almost 100% 4.

The first isolation of HIV-1 was achieved in 1983 by Barré-Sinoussi and collaborators at the Pasteur Institute in Paris 5. Since then, there has been a great advance in the knowledge of HIV, being one of the best-characterized known viruses, and a large amount of information has been accumulated about its biology, transmission and pathogenesis.

Although there is no cure for HIV infection, effective antiretroviral therapy (ARTs) can control the virus and help prevent transmission to other people. At the end of 2018, approximately 79% of people living with HIV knew their status, 62% were receiving antiretroviral therapy (ART) and 53% had achieved suppression of the HIV with no risk of infecting others. Between 2000 and 2018, new HIV infections fell by 37% and HIV-related deaths fell by 45%, with 13.6 million lives saved due to ART 4.

In the case of SARS-CoV-2, the first cases appeared in December 2019 in Wuhan, in the Chinese province of Hubei. There were 41 cases of people with pneumonia, 66% of which had direct exposure in a seafood market where all kinds of meat and fish were sold, so it is thought that the origin might be there. The first news were released in January 2020 when the complete viral genome sequence was published 6, showing that it was a new coronavirus belonging to the same group as the coronavirus related to the severe acute respiratory syndrome (SARS-CoV) causing the outbreak from SARS 2003 7-9.

On March 11^th^, when globally confirmed cases exceeded 118,000 and the number of deaths were 4,291, the WHO characterized the SARS-CoV-2 infection as a pandemic named COronaVIrus Disease 19 (COVID-19) 4. From that day on, most countries in the world have suffered the infection and it is not yet controlled.

## Material and methods

This review provides a comprehensive overview of HIV and SARS-CoV-2 viruses similarities and differences. The data source was PubMed and the search terms and strategy were focused on the definition, epidemiology, transmission mechanism, symptomatology and finally treatment and vaccine development. The results are summarized in a narrative manner.

## Results and Discussion

### Human Immunodeficiency Virus (HIV)

#### Definition

HIV is a virus that belongs to the genus *Lentivirus*, subfamily *Orthoretrovirinae*, family *Retroviridae*. Two types have been identified, HIV-1 and HIV-2 10.

The HIV-1 virion is a spherical particle, with around 100 nm of diameter, that contains two copies of single-stranded RNA together with the enzymatic machinery (reverse transcriptase and integrase) implicated in its transformation from RNA to DNA in the cytoplasm of the host cell and the subsequent integration of this material into the cell genome (proviral DNA) 11. The HIV-1 envelope is formed by viral gp41 and gp120 envelope glycoproteins, with ability to bind to the CD4 surface protein, present in certain cells, including T lymphocytes and mononuclear phagocytic cells. This union produces a conformational change in the viral glycoprotein that allows its subsequent interaction with one of the chemokine receptors (CCR5 and CXCR4), which act as co-receptors for the virus, allowing its entry into the cell. Once inside, it will use the enzyme reverse transcriptase to convert its RNA into DNA, which will be later transported to the nucleus and integrated into the cellular DNA. As mentioned above, HIV-1 infects immune system key cells, mainly CD4+ T helper lymphocytes. Consequently, there is a depression of the immune system. If this depression becomes chronic, the patient progresses to AIDS 12.

#### HIV epidemiology

According to UNAIDS (United Nations on HIV/AIDS) in its most recent update in 2018, there are 37.9 million estimated individuals living with HIV all over the world. There are estimations that predict 1.8 million of new HIV-1 infections each year. The results show that new infections (of all ages) decreased from a peak of 3.4 million in 1996 to 1.8 million in 2017 13. From the start of the pandemic until 2018, 32 million people died from AIDS-related illnesses 13.

#### HIV transmission mechanism

There are three transmission mechanisms of HIV-1 infection: sexual, parenteral and vertical 14. HIV is found in blood, pre-seminal fluid, semen, vaginal fluids, and breast milk, and is transmitted through direct contact of these fluids with the mucosa or bloodstream of another person.

According to the data available, in Spain, the most frequent transmission mechanism nowadays is sexual, mainly from men who have sexual relations with other men (approximately 54.3% of all new cases), followed by heterosexual transmission (28%) and injecting drug use (less than 3.1%) 12.

#### HIV symptomatology

The symptoms generated by HIV infection begin to appear between 2 and 6 weeks after contact with the virus and can be divided into early infection (within the first two months after infection) or chronic. During the early or acute phase of infection, infected people present fever, headache, muscle pain, rashes, sore throat and mouth sores, and swollen lymph nodes. These symptoms can be so mild that are almost not noticed 5.

In the phase of chronic infection, the virus continues spreading and destroying immune cells, causing immunosuppression. Usually, if it is not treated, HIV turns into AIDS in an average of 10 years. When AIDS occurs, the immune system is already severely damaged, opportunistic infections, neurodegenerative diseases and cancers occur in infected individuals 5.

#### Treatment and vaccine development

After the initial discovery of some drugs that had shown high toxicity and scarce benefit (zidovudine, didanosine, zalcitabine), in 1995 the discovery of a combination therapy, which initially included antiprotease drugs (indinavir, saquinavir) was such a change that it was possible to significantly reduce mortality. Antiretroviral therapies (ART) have tranformed HIV infection into a chronic disease 12. After 2008, the emergence of new drugs, including integrase inhibitors, has led to significantly less toxicity and excellent tolerance 15.

However, HIV persists in the body due to the early establishment of reservoirs, which cannot be eliminated with any of the current antiretroviral regimens 16. Reservoirs are defined as anatomical sites or cells in which HIV infection is persistent and stable allowing competent viruses to replicate under permissive conditions.

Studies searching for vaccines have not been successful yet.

### SARS-CoV-2

#### Definition

SARS-CoV-2 is a β-coronavirus, from the sub-genus *Sarbecovirus*, subfamily *Orthocoronavirinae*. The size of the SARS-CoV-2 virion is approximately 50 to 200 nm of diameter, and its genome is single-stranded positive-sense RNA. The complete genome of the SARS-CoV-2 virus has been sequenced, it has 29.2 kb 9, with a variable number between 6 and 20 Open Reading Frames (ORFs) 17. Two thirds of the viral RNA are found in the first ORF (ORF1a/b), which is translated into two structural proteins (pp1a and pp1ab) and 16 non-structural proteins (NSP), whereas the other ORFs encode accessory and structural proteins. The rest of the virus genome encodes four essential structural proteins, namely spike glycoprotein (S), small envelope (E), matrix (M) and nucleocapside (N) 18. Additional accessory proteins interfere with the immune response of the host.

Zhou *et al.* 3 confirmed that SARS-CoV-2 uses the ACE2 receptor to enter into the cells, just as SARS-CoV-1 does. This receptor is expressed in the lung, heart, blood vessels, intestine and kidneys 19. The coronavirus membrane glycoprotein S is the one that binds to the ACE2 receptor on the surface of human cells 20. The S glycoprotein has two subunits, namely S1 and S2 21. S1 determines the virus-host ratio and cell tropism with the key fusion domain, which is RBD, while S2 mediates the fusion of the virus cell membrane by two tandem domains: heptad repeats 1 (HR1) 22 and 2 (HR2) 23. After the fusion virus-cell occurs, the virus genome is released into the cytoplasm and the RNA translates two polyproteins, pp1a and pp1ab 24, which encode non-structural proteins and form replication-transcription complexes (RTCs) in double-membrane vesicles 25. RTCs replicate and synthesize sets of subgenomic RNAs 26 that encode for structural and accessory proteins. By using cellular endoplasmic reticulum and the Golgi, newly formed RNAs, nucleocapsid proteins, and envelope proteins bind and form new viral particles 27. Finally, the vesicles containing the virions fuse with the cell membrane and the virus goes out to infect new cells. The difference with HIV is that SARS-CoV-2 does not integrate in the host DNA. The generation of antibodies 15 days after infection is 100%, both in mild and severe cases 28-32.

#### SARS-CoV-2 epidemiology

The situation of SARS-CoV-2 infection today, November 7th 2020, according to the WHO, is as follows: 48,534,508 confirmed cases worldwide with 1,231,017 deaths since the start of the pandemic in December 2019. Currently, the continent with most cases is America with 21,168,405 confirmed cases and 650,705 deaths. In Europe, the confirmed cases are 12,490,012 with 303,707 deaths 4.

The mortality rate of SARS-CoV-2 (3.8%) is lower than that of SARS-CoV-1 or MERS-CoV (Middle East Respiratory Syndrome Coronavirus), whose rates were 10% and 37.1% respectively, but the number of cases of infection is 10 times higher. This is due to the fact that SARS-CoV-2 can be transmitted from people with no symptoms or with mild infections. These characteristics can explain the sudden pandemic spread of the virus 33. In general the mortality rate is 5% 34, being 49% in critical cases 35, 36.

#### SARS-CoV-2 transmission mechanism

The virus is transmitted through the air, mainly due to small drops of saliva from infected people by coughing or sneezing that can reach two meters 37. The transmission is also produced by direct contact with these secretions or by objects contaminated by them 4.

#### SARS-CoV-2 symptomatology

The symptoms generated by SARS-CoV-2 begin to appear between 2 and 14 days after the contact with the virus. The most common symptoms include fever, cough, and dyspnea. Diarrhea and abdominal pain are also frequent. Although most cases have mild symptoms, in the most severe cases, the infection can cause pneumonia, severe difficulty breathing, kidney failure, and even death 38, 39.

#### Treatment and vaccine development

COVID-19 treatment initially included drugs previously used in HIV treatment, such as lopinavir; subsequent studies demonstrated its lack of efficacy 40. Moreover, as in the case of HIV infection, the first antiviral used (Remdesivir) has shown very limited efficacy 41, 42. Apart from corticosteroids to decrease cytokine storm 43, there are no other useful drugs against SARS-CoV-2 infection.

Many trials are being done to find a vaccine against SARS-CoV-2 44, some of them with encouraging results in phase II.

### Differences between HIV-1 and SARS-CoV-2

Table 1 shows a summary of the main differences described between both viruses.

### Similarities between both viruses

Even though it may seem that these two viruses induced diseases are not very similar among them, they have some important points in common:

(a) Fear in the population. HIV can affect anyone, independently of their social status, race, gender, etc. This can affect people psychologically, making them feel fear, stress or anxiety. Apart from those factors, in COVID-19, there are others that can make people feel this - the virus is new, there are not known effective antivirals, the disease is more contagious than expected, it can even severely affect young individuals with no previous pathologies, respiratory failure forces hospitalization for many days… In addition, the panic is even increased by the presence of the Internet, over-information, spreading of unfounded rumors, and hyper connectivity in our lives nowadays.

(b) Existence of animal reservoirs. The existence of natural animal reservoirs is another point in common, although they are found in different animals, being non-human primates in the case of HIV, and bats in the case of SARS-CoV-2.

(c) Increased synthesis of proinflammatory cytokines. Both viruses generate an increase in the production of cytokine, and this is linked to the viral load in the case of SARS-CoV-2. These cytokines are related with secondary complications in infected people.

It is well known that in HIV infection, the cytokine release is a chronic mechanism that generates prolonged inflammation. Sustained inflammatory status has been related with increased intestinal permeability and bacterial translocation, detected in HIV-infected patients. Intestinal permeability, bacterial translocation or systemic inflammation cannot be reversed with antiretroviral therapies 45. Residual viral replication and other co-infections also contribute to prolonged inflammatory status 45. Serum levels of the proinflammatory interleukin 6 has been independently associated to morbidity (cardiovascular disease, cancer, etc.) and mortality in patients with controlled HIV replication 46.

Regarding COVID-19, the cytokine secretion is an acute response and is implicated in clinical manifestations. Proinflammatory cytokines and chemokines attract more inflammatory cells to migrate from the blood, inducing an amplification of the deleterious response; in fact, a substantial part of the therapy is aimed at blocking pro-inflammatory cytokines 47. Cytokine storm is implicated in the acute respiratory distress syndrome or multiple-organ dysfunction. Increased markers of systemic inflammation are prognostic variables in COVID-19 48.

(d) Modifications of the intestinal microbiota. It has been proved that patients infected with SARS-CoV-2 who develop cardiac complications have higher levels of intestinal permeability and activation of inflammasomes, suggesting a heart-intestine axis in COVID-19 49. In fact, one of the vaccines being developed is a genetically modified probiotic with a plasmid that contains the DNA of the SARS-CoV-2 protein S (NCT04334980).

HIV infection has an unfavorable effect on the interaction between the commensal microbiota and the immune system 50. Microbiota modifications (increase in pro-inflammatory bacteria and a reduction in those that promote homeostasis) have been detected in HIV infected people with pathogenic consequences on bacterial translocation (see above) and immune responses 51-61. It is important to mention that elite controllers have microbiomes more similar to those of healthy individuals than other groups of HIV-infected patients 62, and they also show lower levels of immune activation and HIV reservoirs 63.

(e) Neutrophil extracellular traps (NETs) formation. The two viruses share a mechanism known as NETosis. This is a neutrophil death mechanism in which neutrophils release nets of chromatin fibers into the extracellular space. Those nets contain histones, microbicidal peptides or oxidizing enzymes 64. NETs are very adherent and capture extracellular microbes, such as some bacteria, fungi and virus, stimulating its removal 64-66. Acute NETosis against infectious agents is an efficient defense mechanism that avoids collateral tissue damage, concentrating the antimicrobial action and reducing the toxicity attributable to the proteases. However, a chronic or acute aberrant NETosis may contribute to the pathology 67. In HIV infection, NETosis has been suggested to be involved in the atherosclerosis development 66. In COVID-19, the virus-induced NETs can circulate in an uncontrolled way, giving rise to an extreme systemic response of the body, increasing the concentrations of cytokines, chemokines and increasing inflammation. In addition to promoting the cytokine storm, NETs in COVID-19 patients are also responsible for the thrombotic complications they present, as is the case in HIV patients 68.

## Conclusion

As a conclusion, we would like to point that although at first sight these viruses do not resemble each other, the molecular mechanisms used are common: the increased of pro-inflammatory cytokine synthesis, the modifications in intestinal microbiota, the NETs formation. Basic science has been trying to understand these mechanisms for years and the greater the knowledge, the lesser the damage to the population in this pandemic and in the future.

## Figures and Tables

**Table 1:** Differences between HIV and SARS-CoV-2

	HIV	SARS-CoV-2
Phylogenetically	*Lentivirus*	β-coronavirus
Transmission mechanism	Sexual, parenteral and vertical direct contact.	Air by small drops of saliva.
Targets	CD4 surface protein expressed in CD4+ T helper lymphocytes, macrophages, and dendritic cells.	ACE2 receptor expressed in lung, heart, blood vessels, intestine, and kidney.
Symptoms	In early infection: flu-like and/or mononucleosis-like symptoms; in chronic infection: opportunistic entities; usually, if it is not treated, HIV turns into AIDS in about 10 years.	Difficulty breathing and fever. In the most severe cases, the infection can cause pneumonia, severe difficulty breathing, kidney failure, and even death.
Days from infection with symptoms	Between 2 and 6 weeks after contact with the virus.	Between 2 and 14 days after contact with the virus.
Death percentage without treatment	≥95%	1-4%
